# Whole-Genome Resequencing Reveals the Diversity of Patchouli Germplasm

**DOI:** 10.3390/ijms241310970

**Published:** 2023-06-30

**Authors:** Zhipeng Li, Yiqiong Chen, Yangyan Li, Ying Zeng, Wanying Li, Xiaona Ma, Lili Huang, Yanting Shen

**Affiliations:** 1 Institute of Medicinal Plant Physiology and Ecology, School of Pharmaceutical Sciences, Guangzhou University of Chinese Medicine, Guangzhou 510000, China; 20201110602@stu.gzucm.edu.cn (Z.L.); chenyq0828@gmail.com (Y.C.); 20211110141@stu.gzucm.edu.cn (Y.L.); 20212110036@stu.gzucm.edu.cn (Y.Z.); liwy2@chinaconsun.com (W.L.); insider1016@gmail.com (X.M.); lilihuang@gzucm.edu.cn (L.H.); 2 State Key Laboratory of Plant Cell and Chromosome Engineering, Institute of Genetics and Developmental Biology, Chinese Academy of Sciences, Beijing 100000, China

**Keywords:** patchouli, whole-genome resequencing, genetic variation

## Abstract

As an important medicinal and aromatic plant, patchouli is distributed throughout most of Asia. However, current research on patchouli’s genetic diversity is limited and lacks genome-wide studies. Here, we have collected seven representative patchouli accessions from different localities and performed whole-genome resequencing on them. In total, 402,650 single nucleotide polymorphisms (SNPs) and 153,233 insertions/deletions (INDELs) were detected. Based on these abundant genetic variants, patchouli accessions were primarily classified into the Chinese group and the Southeast Asian group. However, the accession SP (Shipai) collected from China formed a distinct subgroup within the Southeast Asian group. As SP has been used as a genuine herb in traditional Chinese medicine, its unique molecular markers have been subsequently screened and verified. For 26,144 specific SNPs and 16,289 specific INDELs in SP, 10 of them were validated using Polymerase Chain Reaction (PCR) following three different approaches. Further, we analyzed the effects of total genetic variants on genes involved in the sesquiterpene synthesis pathway, which produce the primary phytochemical compounds found in patchouli. Eight genes were ultimately investigated and a gene encoding nerolidol synthetase (*PatNES*) was chosen and confirmed through biochemical assay. In accession YN, genetic variants in *PatNES* led to a loss of synthetase activity. Our results provide valuable information for understanding the diversity of patchouli germplasm resources.

## 1. Introduction

As a perennial plant from Lamiaceae family, patchouli (*Pogostemon cablin* (Blanco) Benth.) is a traditional Chinese medicinal herb whose aerial parts are used for medicinal purposes. It is known for its ability to dispel moisture, provide relief from summer heat and exterior syndrome, and act as an anti-vomiting agent in the traditional Chinese medicine system [1]. The essential oils extracted from patchouli have a variety of beneficial activities, including anti-inflammatory, antioxidant, anti-ulcer, anti-colitis, anti-mucositis, and protective effects against brain and lung damage [2,3,4,5,6,7]. In addition, patchouli essential oils are also widely used in the light chemical industry, such as the production of various agents, insecticides, fragrance fixatives, cosmetics, and skin care products [8,9]. Given the important medicinal and economic values, the global demand for patchouli oil is >1600 tons per year [10].

Patchouli originates from Southeast Asia, such as Indonesia and Philippines [11]. Since its introduction to China, patchouli has been extensively cultivated in Guangdong and Hainan provinces. Among them, Shipai patchouli from Guangdong province has been regarded as the genuine herb in traditional Chinese medicine due to its superior medicinal effect and quality. Previous studies have shown that patchouli from different localities varies in phenotypic traits and volatile oil compositions [12,13,14,15]. Technologies such as Random Amplified Polymorphic DNA (RAPD) [16], Sequence-Related Amplified Polymorphism (SRAP) [17], and Specific-Locus Amplified Fragment (SLAF) [18] have been utilized to identify polymorphic genetic markers in patchouli germplasm resources. However, these analyses were biased towards simple amplification of genetic markers, involved fewer genetic loci, and the results are limited. To date, there has been a dearth of genome-wide research on the genetic diversity of patchouli.

The primary bioactive ingredients of patchouli oil are sesquiterpenoids [19]. As an important metabolite, sesquiterpenoids are synthesized through the mevalonate (MVA) and methylerythritol phosphate (MEP) pathways, which produce the isopentenyl diphosphate (IPP) [20] and dimethylallyl diphosphate (DMAPP) [21]. IPP and DMAPP were subsequently converted into different sesquiterpenes by sesquiterpene synthases. While the sesquiterpene synthesis pathway of patchouli has been fully resolved and relevant studies have dissected the significant expression differences of sesquiterpene synthesis genes in patchouli from different localities [22,23], the genetic diversity of these genes and their contribution to the diversity of patchouli metabolites have not yet been investigated.

As an important component of the diversity of germplasm resources, genetic variation is widely present in plants and indirectly affects plant phenotype and metabolite synthesis by influencing gene function. For instance, point mutations and small deletions in *BrTT1* were found between yellow-seeded and brown-seeded Dahuang plants [24]. As a medicinal plant that is widely cultivated in Asia, patchouli plants from different regions exhibit phenotypic and metabolite differences, which can be attributed to genetic variations at the DNA level. Performing an analysis of genes affected by genetic variation could provide a deeper understanding of the causes of patchouli germplasm diversity.

With the rapid development of high-throughput sequencing technology, the recent release of the chromosome-level patchouli genome [25] has enabled us to analyze the genetic diversity among patchouli accessions at the genome-wide level. In this study, whole-genome resequencing was conducted on several patchouli accessions. The objectives of our study: (i) To uncover the genetic diversity among representative patchouli accessions; (ii) To redefine the phylogenetic relationships of patchouli; and (iii) To analyze the effects of genetic variation on metabolite biosynthesis. Furthermore, we tried to create distinctive molecular markers for genuine herb SP patchouli, which could greatly facilitate the cultivation of patchouli.

## 2. Results

### 2.1. Whole-Genome Resequencing of Seven Representative Patchouli Accessions

**Figure 1:**
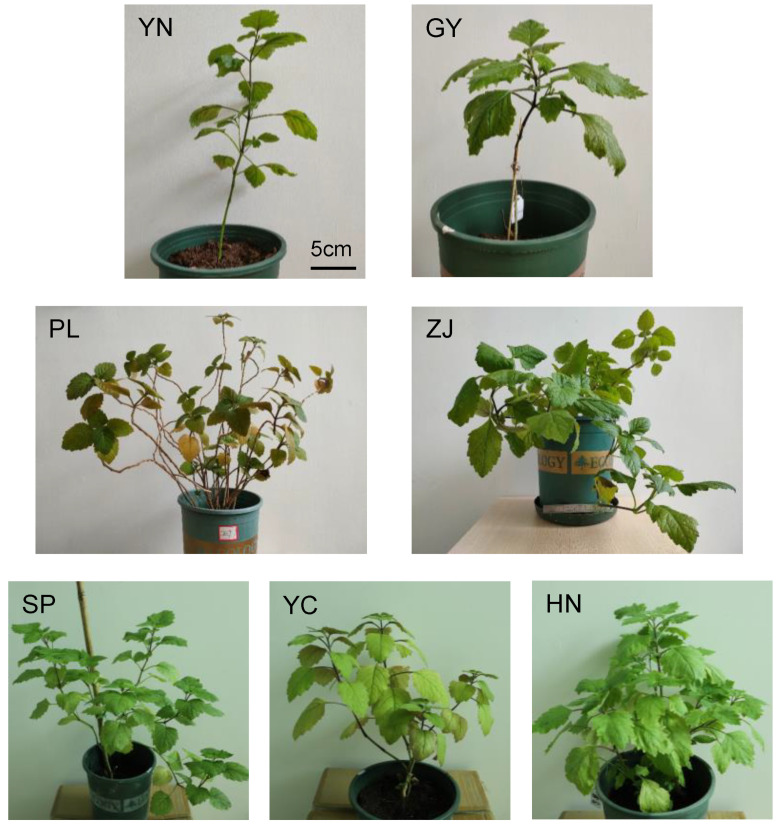
Patchouli accessions from different localities. Different IDs represent patchouli from different localities, among which (**YC**) is patchouli from Yangchun, (**ZJ**) is patchouli from Zhanjiang, (**HN**) is patchouli from Hainan, (**GY**) is patchouli from Gaoyao, (**PL**) is patchouli from Philippine, (**YN**) is patchouli from Indonesia, and (**SP**) is patchouli from Shipai.

A total of seven patchouli accessions from different localities were collected for this study. Among them, there are four accessions from different planting regions in Guangdong province (ZJ, YC, GY, SP), one accession from the main planting region of Hainan province (HN), and two accessions from Southeast Asia (PL, YN) (Figure 1).

Whole-genome resequencing was performed on seven patchouli accessions, resulting in a total of 283.81 Gb of raw reads. After filtering, we have obtained a total of 281.60 Gb of clean reads. All of the clean reads were aligned to the reference genome. Based on the mapping results, the average depth was calculated to be 21.5× (Table 1). The data processing results showed that the patchouli resequencing data has a coverage of over 99% on the reference genome, with an average coverage of 99.54%, which means the saturation of our data. At the same time, we also calculated the mapping rate of each accession to the reference genome and found that all of them were above 94%, indicating low contamination among the sequenced accessions. However, differences exist among the mapping rate of different accessions while the lowest and highest mapping rates are 94.56% and 99.87%, respectively, indicating that there may be variations between accessions, which could affect the diversity of patchouli germplasm resources.

### 2.2. Massive Genetic Variants Exist among Patchouli Accessions

By performing the genetic variation detection pipeline shown in Supplementary Figure S1, we detected a total of 30,079,205 raw genetic variant loci from seven patchouli accessions, including SNPs and INDELs. To account for the possibility of false discovery of variations, we applied additional filters to the genetic variants based on the criteria as follows: QD < 2.0, MQ < 40.0, FS > 60.0, SOR > 3.0, MQRankSum < −12.5, and ReadPosRankSum < −8.0. We finally obtained 402,650 high-quality SNPs and 153,233 high-quality INDELs (Table 1).

**Figure 2:**
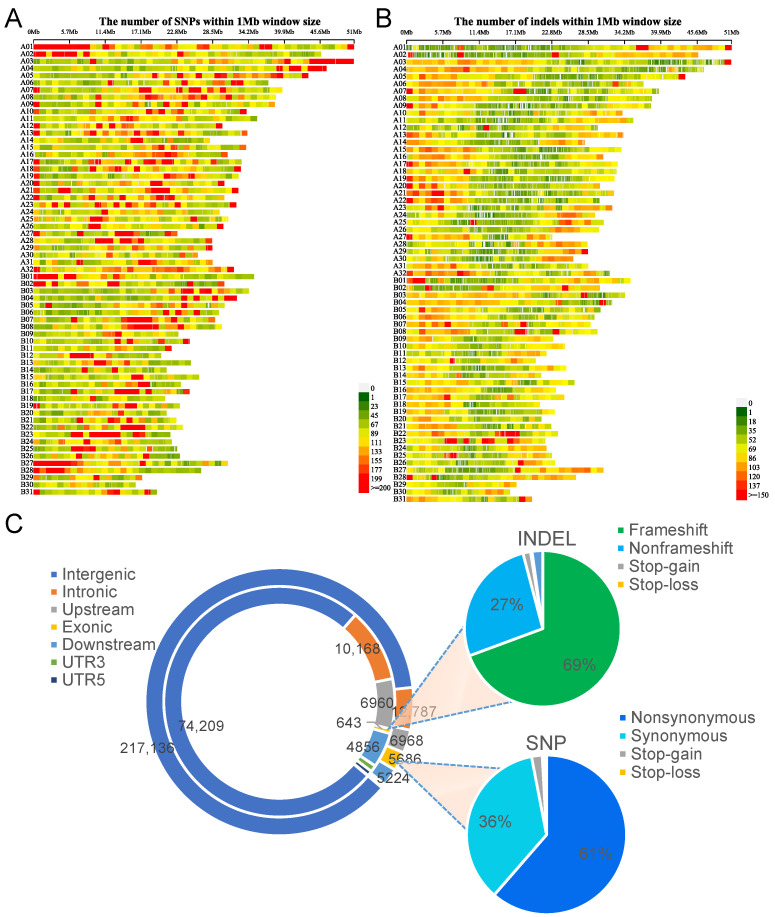
Distribution and annotation for SNPs and INDELs. (**A**,**B**) Density distribution of all SNPs (**A**) and INDELs (**B**) on chromosomes; (**C**) Genome annotation and functional annotation of SNPs and INDELs. The left doughnut diagram is the result of the genome annotation, the outer circle is the SNP, and the inner circle is INDEL. The right pie chart is the functional annotation for INDEL (top) and SNP (bottom).

We further analyzed the distribution of variants on chromosomes and observed that SNPs were predominantly located in the middle of most chromosomes, with the exception of chromosomes A01, A02, B27, and B28, where the SNPs were concentrated at the ends of the chromosomes (Figure 2A). On the other hand, INDELs were primarily located at the ends of most chromosomes, with the exception of B22 and B23 chromosomes, where INDELs were concentrated in the middle region (Figure 2B). The statistics on the length of INDELs showed that the majority of them were within 5 base pairs (bp) (Supplementary Figure S2), which is consistent with the findings of previous studies [26,27]. In further, we found that the number of SNPs and INDELs differs significantly among different patchouli accessions. PL has the highest number of SNPs (250,663) and INDELs (73,501), whereas ZJ has the lowest number of SNPs (95,858) and INDELs (29,126) (Table 1). Furthermore, the number of genetic variants in HN, YC, and GY were similar to ZJ (around 106 k for SNPs and 32 k for INDELs), while YN and SP were similar to PL (around 249 k for SNPs and 65 k for INDELs). This suggests that the seven patchouli accessions may be divided into two groups.

The SNPs and INDELs that may affect gene function were then examined. They were initially grouped into seven clusters based on their position relative to the annotated transcripts in the patchouli genome (the circle panel in Figure 2C). The SNPs and INDELs located in the exonic region were analyzed to determine their impact on the proteins they encode (the pie charts in Figure 2C). Among all SNPs, 2.29%, 5.16%, 2.81%, 2.10%, and 87.62% of the variants were located in the exon, intron, upstream, downstream, and intergenic regions, respectively (Figure 2C). Among the SNPs located at coding region, we annotated 3490 nonsynonymous, 123 stop-gain, and 31 stop-loss mutations (Figure 2C). These mutations have the potential to cause changes in the amino acid. In addition, 0.66%, 10.50%, 7.18%, 5.01%, and 76.63% of INDELs were located in the exon, intron, upstream, downstream, and intergenic regions, respectively. A total of 441 frameshift, 10 stop-gain, and two stop-loss mutations were annotated in the coding region (Figure 2C).

### 2.3. Patchouli Accessions Can Be Divided into Three Groups

**Figure 3:**
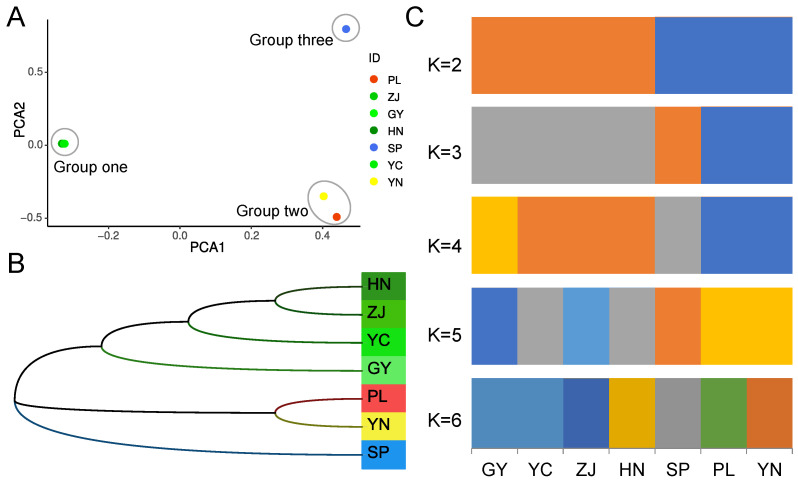
Phylogenetic relationship analysis. (**A**) Principal component analysis on seven patchouli accessions. (**B**) Neighbor-joining phylogenetic tree-based analysis on all high-quality SNPs. (**C**) Population structure analysis of patchouli from different sources using FRAPPE.

We determined the phylogenetic relationships among the seven patchouli accessions by utilizing a combination of principal component analysis (PCA), phylogenetic tree construction, and stacked plot of population structure. Based on PCA, the first principal component (PC1) separates the accessions SP, YN, and PL from the other four accessions, while the second principal component (PC2) separates SP from YN and PL. Therefore, the seven patchouli accessions were clearly divided into three groups. Group one includes HN, ZJ, GY, and YC. Group two consists of PL and YN, while the third group is represented solely by SP (Figure 3A). The first two groups consisted of individuals from different regions. Group one comprised solely of individuals from China, while group two consisted of individuals from Southeast Asia. Consistent with the results of principal component analysis (PCA), the phylogenetic tree also divided the seven patchouli accessions into three groups (Figure 3B). For the stacked plot of population structure, the seven patchouli accessions were initially divided into two groups when K = 2: HN, ZJ, YC, GY, and SP, PL, YN. The SP accession was then separated into its own group when K = 3 (Figure 3C). Calculation of cross-validation error showed that when K = 3, the cross-validation error value is minimal. So, the population division of the seven patchouli accessions was consistent with the results of the PCA and phylogenetic tree (Figure 3C). Based on the aforementioned results, we categorized the seven patchouli accessions into three distinct groups: the Chinese group (HN, ZJ, YC, GY), the Southeast Asian group (PL, YN), and SP, which formed a separate group from the Southeast Asian group.

As SP patchouli is currently planted specifically in Shipai village, Tangxia village, and other nearby areas of Guangzhou city in Guangdong province [28], we speculate that this particular variety of patchouli may have been introduced from Southeast Asia at a specific stage than other patchouli accessions in China. After undergoing artificial breeding and being influenced by the environment, it has become a genuine herb in Chinese traditional medicine.

### 2.4. Detection and Verification of Molecular Markers for the Genuine Patchouli SP

**Figure 4:**
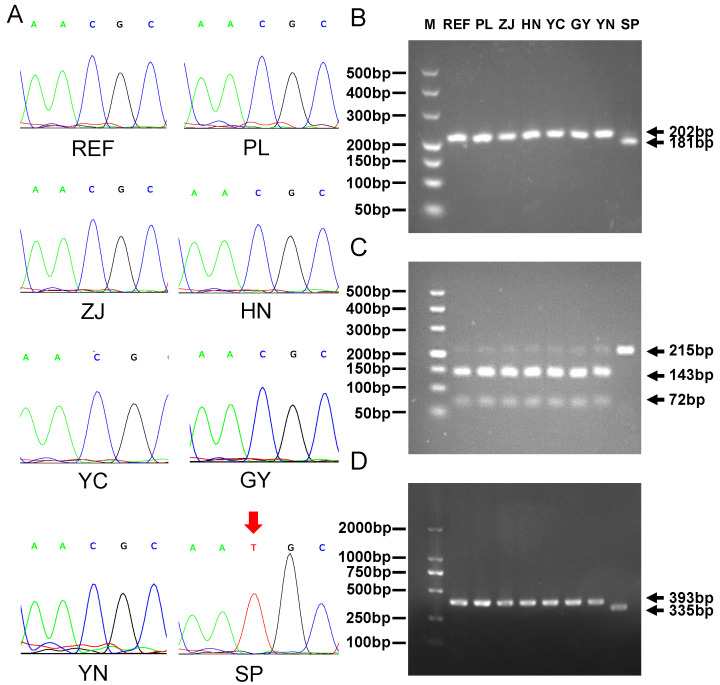
Verification of SP specific molecular markers. (**A**) Sanger sequencing verification for SNP mutation of A24-2156111. The arrow is the location of the SNP location. (**B**,**C**) Enzymatic digestion verification for SNP of A07-9038661 (**B**) and B23-13365305 (**C**). (**D**) PCR directly amplifies the sequence for INDEL mutation of A01-27218088; REF represents the sequence of reference patchouli genome.

In this study, we utilized genetic variants to screen 26,144 homozygous SNPs and 16,289 homozygous INDELs as molecular markers for SP patchouli. These markers were selected due to their distinct genotypes in SP compared to the other six patchouli accessions. These variants are abundantly distributed on chromosomes other than A32 (Supplementary Figure S3). To confirm the accuracy of these variants, five SNPs and five INDELs were randomly selected and verified using Polymerase Chain Reaction (PCR) following gel electrophoresis, Sanger sequencing, and restriction enzymatic digestion (Table S1). The patchouli genome, which was released in a previous study [25], was used as a reference in this study. As shown in Figure 4A and Supplementary Figure S4A, the SNPs A24-2156111 and A20-14848597 were validated using PCR following Sanger sequencing. The sequence peak showed that mutations were only present in the SP accession, and the type of mutation is consistent with SNP mutation. The SNPs A07-9038661, B23-13365305, and A02-38522213 were verified through restriction enzyme digestion (Table S1). When the primer forms a digestion site with the mutation (A07-9038661), the band of the SP accession after digestion was approximately 21 base pairs shorter than the other patchouli bands (Figure 4B). As the digestion site is located on the unmutated sequence (B23-13365305), all patchouli samples, except for the SP accession, were cleaved into multiple bands (Figure 4C). On the other hand, since the digestion site is located on the mutant sequence (A02-38522213), the SP accession was cleaved into multiple bands (Supplementary Figure S4B). The five INDEL markers, namely, A01-27218088, A15-14220318, B04-23072200, B09-16858306, and B22-12443436, were validated through PCR following 2% concentrated agarose gel electrophoresis. The bands show that INDEL variations were only present in the SP accession (Figure 4D and Supplementary Figure S4C–F). All experimental results show that the mutations were real and unique to Shipai patchouli. This confirms the reliability of the molecular markers of Shipai patchouli we detected, which can be used in the identification of genuine patchouli and the improvement of patchouli germplasm.

### 2.5. Genetic Variations Alter the Protein Structure of Sesquiterpene Synthesis Gene

As mentioned above, genetic variations can affect the amino acids that a gene codes for, which may alter the three-dimensional structure of proteins and ultimately affect the gene function. As sesquiterpene is the main active ingredients of patchouli, we further predict the effect of genetic variation on protein function for genes involved in sesquiterpene synthesis. Based on the annotation of genetic variants, we found that 11,436 genes may be affected by SNP/INDELs. Comparing these genes to the 369 sesquiterpene biosynthetic pathway genes annotated in a previous study [25], we discovered that 46 of them may be influenced by genetic variation, and eight of them contained nonsynonymous mutations in their exon regions (Supplementary Figures S5 and S6). Among these eight genes, *Pat_B10G069300* encodes 3-hydroxy-3-methylglutaryl-CoA reductase (HMGR), *Pat_B15G003400* encodes isopentenyl diphosphate isomerase (IDI), and *Pat_A04G103400* encodes farnesyl diphosphate synthase (FPPS), while the remaining five genes encode terpene synthase (TPS). We initially compared the position of genetic variations with conserved domains for each gene. As a result, we identified four genes (*Pat_A04G080600*, *Pat_A04G103400*, *Pat_B27G086400*, *Pat_B23G065900*) that had mutations located closer to their conserved domains (Table S2). While the other three genes are only affected by one or two variants, *Pat_B23G065900* has 14 nonsynonymous SNP mutations and two INDEL variants, indicating that the function of this gene may be more significantly impacted.

The three-dimensional structure and binding pockets of the proteins encoded by these four genes were predicted (Supplementary Figure S7). For *Pat_A04G080600*, *Pat_A04G103400,* and *Pat_B27G086400*, no significant changes were observed on the prediction of reference and mutated protein structure (Supplementary Figure S7A–C). The shape and size of the binding pockets are similar (Supplementary Figure S7E–G,I–K), and the likelihood of gene function being affected is very low. However, there are differences in the three-dimensional protein structure of *Pat_B23G065900*. As shown in the Supplementary Figure S7D, the mutations are mainly located at the end of the sequences, which results in an irregularly curved shape with no overlapping areas. In the predicted binding pockets of this protein, 11 out of the 16 mutations were located. These mutations included 2 INDELs and 9 SNPs, indicating that the gene mutation may have a significant impact on the protein structure (Supplementary Figure S7H,L). Upon further comparison of the binding pockets between the reference and mutational proteins, it was observed that the binding pocket had significantly reduced in size after mutations. This suggests that SNP and INDEL mutations could potentially affect the function of this protein. Due to the significant shrinking of the protein-binding pocket, we suspect that genetic variations in *Pat_B23G065900* could alter its function.

### 2.6. Genetic Variants in PatNES Cause the Loss of Nerolidol Synthetase Activity in Patchouli Accessions from Southeast Asia and SP

As a terpene synthase gene, *Pat_B23G065900* has the biological activity of catalyzing farnesyl pyrophosphate (FPP) to generate sesquiterpene [29,30]. However, it is not clear which sesquiterpene it produces, and the effect of the mutation on gene function also needs to be explored.

**Figure 5:**
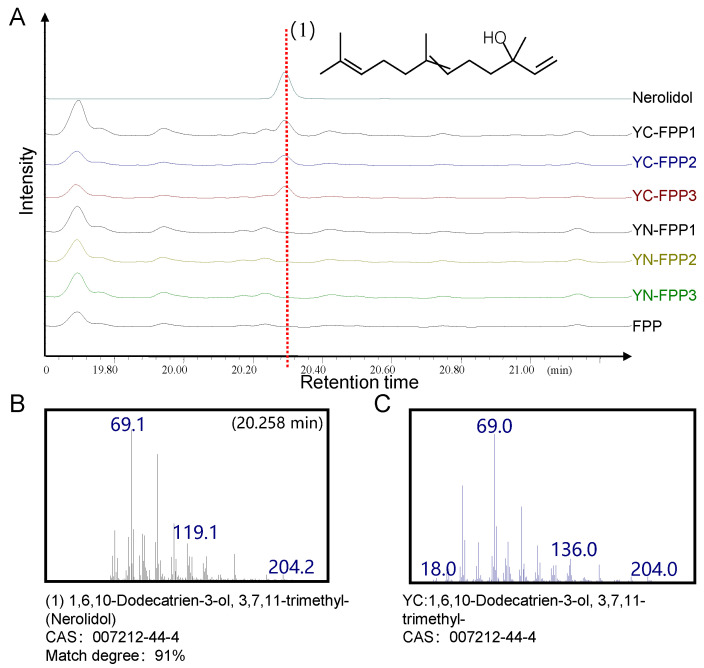
Biochemical assay of *PatNES*. (**A**) GC-MS detects in vitro enzymatic reaction products of YC and YN. Mass spectra of standard nerolidol (**B**) and products of YC (**C**).

Based on the detection of variants in *Pat_B23G065900* (Table S2), patchouli accessions from Southeast Asia (PL, YN) and SP shared the same mutations, while the Chinese accessions did not show any mutations (Supplementary Figure S8). To investigate the impact of mutations on gene function, we employed in vitro enzyme activation to assess the effect of mutations on *Pat_B23G065900,* using YC and YN as reference and mutation models. After cloning, the coding sequences of *Pat_B23G065900* in both accessions, expressing the proteins they encoded in *E. coli*, were purified. We subsequently used FPP as the substrate and purified protein as the catalyst to conduct an in vitro enzymatic reaction. The products were detected using Gas Chromatography-Mass Spectrometry (GC-MS). A chromatographic peak appeared in YC at 20.258 min, while nothing was detected in YN (Figure 5A). The retention time of this peak was consistent with the chromatographic peak retention time of the standard nerolidol, and their match degree was 91% (Figure 5B,C). The enzymatic reaction product of *Pat_B23G065900* is nerolidol, indicating that the gene is a nerolidol synthase gene (*PatNES*). These results indicate that genetic variations in *PatNES* among the patchouli accessions in Southeast Asia and SP inactivate its nerolidol synthetase function. This may lead to a diversity of downstream metabolites of nerolidol among patchouli accessions.

## 3. Discussion

Genetic variation plays a crucial role in maintaining biodiversity, and it deepens our understanding of the germplasm resource diversity. Benefiting from high-throughput and low-cost whole-genome resequencing is currently the most commonly used method for detecting genetic variation. With the application of whole-genome resequencing in different medicinal plants, we now have a more comprehensive understanding of their genetic diversity [31,32]. Here, we conducted the whole-genome resequencing of the medicinal herb patchouli and detected large number of genetic variants. As far as we know, it is the first genome-wide detection of genetic variation for patchouli.

Based on the high-quality SNPs and INDELs detected through whole-genome resequencing, we were able to deduce the genetic composition of patchouli accessions. By analyzing the population structure, seven representative patchouli accessions were divided into three groups: the Chinese group (HN, ZJ, YC, and GY), the Southeast Asia group (PL and YN), and SP. However, in previous research, different methods have resulted in varying population structure segmentations. For instance, Pan et al. [16] detected 84 polymorphic DNA fragments based on random amplified polymorphic DNA (RAPD) technology and separated five patchouli accessions into two groups. Among these SP, GY, and HN patchouli formed a cluster, while ZJ formed a branch with one patchouli from Guangzhou city. Using sequence-related amplified polymorphism technology (SRAP), Wu et al. [17] amplified 255 polymorphic DNA fragments from 16 patchouli accessions and classified them into two distinct groups. Group one includes five accessions from Hainan Province (HN) and six accessions from Zhanjiang city (ZJ), while group two comprises three accessions from Gaoyao City (GY) and two accessions from Guangzhou City. Huang et al. [18] further explored the relationship between 22 patchouli accessions with Specific-Locus Amplified Fragment (SLAF) technology, resulting in the detection of 511 SNPs. Based on these reliable SNPs, the patchouli accessions were divided into six distinct groups: one group from Vietnam, two groups from China, and three groups from Indonesia. Due to the discrepancies in these phylogenetic results, it is hard to reach a consensus on the introduction and spread history of patchouli, especially those planted in China. In this study, which utilized whole-genome resequencing technology, genetic variations in patchouli accessions were detected at the whole-genome level. A total of 402,650 SNPs were used to perform a phylogenetic analysis. By combining PCA, phylogenetic tree, and stack plots of population structure, we have demonstrated that the majority of patchouli accessions in China share a close kinship, with the exception of the SP patchouli, which is closer to patchouli accessions in Southeast Asia.

Based on the genetic variants detected by resequencing, 26,144 specific SNPs and 16,289 specific INDELs were screened as molecular markers for SP accession. Out of these, 10 were verified successfully. As the genuine accession for patchouli, SP has the best medicinal properties and quality in Chinese traditional medicine. Combining these SP specific genetic variation to form specific fingerprints, such as developing SNP/INDEL-based chips [33], would be very useful to identify genuine herb of patchouli. Furthermore, SP-specific SNPs and INDELs can be utilized to identify significant functional genes in SP [34], including mutations that are exclusive to certain phenotypes or genes related to metabolite synthesis. By analyzing these unique mutations, we can even further analyze the cause of genuine medicinal herb.

The genes affected by genetic variation in the sesquiterpene synthesis pathway were also explored. The results showed that the *PatNES* was inactivated in the Southeast Asia group and SP. The SNP/INDEL mutations in these accession result in the alteration of 16 amino acids in the *PatNES* protein (Table S2). Notably, mutations in positions 475, 589, 599, and 641 are located closest to the conserved domain of the protein. A protein domain is a distinct region within a protein that possesses a specific spatial structure and independent function. It serves as the fundamental functional unit of the protein, enabling it to carry out its biological functions effectively [35]. Therefore, these mutations are most likely to result in a change in the function of the *PatNES* protein. This hypothesis was further confirmed when we found the three-dimensional structure and binding pocket of the *PatNES* protein were also changed (Supplementary Figure S7). As a nerolidol synthase gene, *PatNES* is responsible for regulating the synthesis of nerolidol in patchouli. However, upon examining the sesquiterpenoids in patchouli, we did not detect any nerolidol (Supplementary Figure S9). Similarly, other studies related to patchouli have also failed to detect nerolidol [19,36]. We believe that this phenomenon may be caused by the fact that nerolidol is not the final product of patchouli metabolism. Instead, it may continue to participate in the next synthetic reaction as an intermediate product, catalyzed by downstream enzymes [37,38].

Functional changes in nerolidol synthase as part of patchouli’s metabolites reveal a reason for the diversity of metabolites in patchouli, i.e., genetic variation affects the final metabolites of plants by influencing the function of metabolic pathway genes. From this perspective, we can interpret more plant metabolites and even agronomic traits diversity. Genetic variation may affect the genes involved in metabolic pathways, thereby altering the final phenotype through changes in metabolite composition and content. Furthermore, the substantial genetic variation found in nature can offer valuable genetic data for the modification of plants, especially medicinal plants, which play an important role in medical therapy.

Whole-genome resequencing has become a popular sequencing technology that has greatly advanced the research on the diversity of germplasm resources of medicinal plants. Due to its ability to detect and analyze genetic variations in medicinal plants at a genome-wide level, this method provides more comprehensive information than RNA-seq and degenerate genome sequencing. This plays a crucial role in understanding population evolution, improving plant varieties, and enhancing the diversity of germplasm resources for medicinal plants. In this study, we reinterpreted the population structure of patchouli by analyzing genetic variation through resequencing. We also developed molecular markers for the genuine herb and analyzed the influence of genes in the sesquiterpene synthesis pathway under the effects of genetic variation. These studies demonstrate that whole-genome resequencing technology could accelerate research on medicinal plants, offer guidance for further medicinal plant research, and alleviate the current lagging situation of medicinal plant research within the traditional Chinese medicine system.

## 4. Materials and Methods

### 4.1. Sample Preparation, DNA Extraction, and Whole-Genome Resequencing

Patchouli samples from various regions were collected for the study. Philippines patchouli (PL) was collected from Yaowang Mountain of Guangzhou University of Chinese Medicine. The other samples, including Gaoyao (GY), Indonesian (YN), Hainan (HN), Shipai (SP), Yangchun (YC), and Zhanjiang patchouli (ZJ), were collected from Professor Ouyang Puyue at Guangdong Food and Drug Vocational College. All of the aforementioned materials are cultivated in the greenhouse at Guangzhou University of Chinese Medicine.

Genomic DNA from different patchouli accessions was extracted from fresh leaves using the Plant Genomic DNA Extraction Kit (DP305, Tiangen, Beijing, China). The quality of the DNA was detected using the NanoPhotometer N50 spectrophotometer (Implen, München, Germany) and 1% agarose gel electrophoresis. Library construction and sequencing were conducted on the Illumina NovaSeq6000 system.

### 4.2. Sequence Processing and Alignment to the Reference Genome

The raw reads were processed using Fastp (v0.20.1) [39] and FastQC (v0.11.9) [40] for quality control. The clean reads were aligned to the patchouli reference genome [25] using the ‘mem’ tool from Burrows–Wheeler Aligner (BWA) (v0.7.17) [41] with default parameters. Samtools (v1.12) [42] was used to convert the mapping results into the BAM format and calculate the mapping ratio. The genome coverage and depth were calculated using Bedtools (v2.30.0) [43].

### 4.3. Genetic Variation Detection and Annotation

The genetic variants (SNP/INDEL) were detected using the Genome Analysis Toolkit (GATK) (v4.2.0.0) [44] and filtered based on the following criteria: QD < 2.0, MQ < 40.0, FS > 60.0, SOR > 3.0, MQRankSum < −12.5, and ReadPosRankSum < −8.0. Then, VCFtools (v0.1.16) [45] were used to further filter the final genetic variation dataset with the following parameters: -max-missing 0.5, --min-alleles 2, --max-alleles 2. The distribution of SNPs and INDELs on each chromosome was visualized with the R package CMplot. The ANNOVAR software (v20200316) [46] was used to annotate the variants in the patchouli genome.

### 4.4. Population Structure Analysis

High-quality SNP datasets detected from seven patchouli accessions were used for analyzing the population structure. Three methods were used to reveal the population structure: principal component analysis (PCA), phylogenetic tree analysis, and stacked plot of population structure. Principal components 1 and 2 were calculated for the SNP dataset using the “--pca” parameter of PLINK (v1.07) [47] and visualized with the ggplot2 package [48] in R (v4.2.3) [49]. A neighbor-joining method was used to construct the phylogenetic tree using the PHYLIP software (v3.697) [50]. The bootstrap value was set to 1000, and the phylogenetic tree was modified on the Itol [51]. The genetic structure of SNP datasets from seven patchouli accessions was performed using FRAPPE software (v1.1) [52], and the number of clusters (K) was considered from 2–6 during the simulation analysis. Calculation of cross-validation (CV) error was conducted using admixture software (v1.3) [53].

### 4.5. The Detection and Verification of Molecular Markers for Patchouli Accession SP

The VariantsToTable subprogram in GATK software (v4.2.0.0) was used to convert the vcf file into a table file; then, the negation function of the awk tool in the Linux system was applied to extract the unique sites of SP patchouli among all genetic variants. Based on the unique mutations of SP patchouli, ten loci were randomly selected for verification. The methodology for enzyme digestion is based on Cleaved Amplified Polymorphic Sequences (CAPS) [54] and Derived Cleaved Amplified Polymorphic Sequences (dCAPS) [55]. The primer containing the digestion site and the restriction enzymes were designed in dCAPS Finder 2.0 (http://helix.wustl.edu/dcaps/dcaps.html (accessed on 29 Novemver 2022)). Another primer was designed in Primer Premier 5 software (v5.0). Both the primers from dCAPS Finder and Primer Premier 5 were used to amplify the DNA fragments containing SNPs. The primer pairs used to amplify fragments containing INDELs were designed in Primer Premier 5 software. The sequences containing SNPs/INDELs were amplified according to the following protocol: DNA 100 ng/μL, forward and reverse primers 1.5 μL each, 2× Prime STAR MAX 25 uL, and ddH_2_O added to 50 μL. The amplified fragments containing SNPs were either sent for first-generation sequencing at Sangon Biotech or enzyme digestion was performed after PCR. The INDELs were verified by directly amplifying the sequences and comparing the length of the band on agarose gel electrophoresis. All of the primers and enzymes are listed in Table S1.

### 4.6. The Prediction of Three-Dimensional Structure and Binding Pockets of Protein

Three-dimensional structure prediction of proteins was conducted with Alphafold (v2.0) [56] on both the reference protein sequences as well as mutated sequences, and the differences in the three-dimensional structure of proteins were then compared on PyMOL (v2.5.5). We continued to predict the binding pocket of proteins on D3Pockets [57] website (https://www.d3pharma.com/D3Pocket/index.php (accessed on 9 September 2021)) based on the Alphafold prediction. The true binding pockets were determined with the conjunction of conservative domain, and the difference of pockets were also compared on PyMOL.

### 4.7. RNA Extraction and Amplification of cDNA

The fresh, young leaves of YC and YN patchouli were used for the validation of the impact of mutations on *Pat_B23G065900*. The total RNA was extracted following the manufacturer’s protocol of the RNAprep Pure Plant Kit (Tiangen Biotech Co., Ltd., Beijing, China). RNA concentration, purity, and integrity were measured using microUV spectrophotometer and 1.2% agarose gel electrophoresis. According to the instructions of the cDNA synthesis kit (Accurate Biotechnology (Hunan) Co., Ltd., Changsha, China), the RNA from patchouli was reverse-transcribed into cDNA.

### 4.8. Cloning of the Full-Length CDS of PatNES

The coding sequences of *Pat_B23G065900* (*PatNES*) in YC and YN were amplified based on the specific primers (Table S3) designed on the Vazyme website (https://crm.vazyme.com/cetool/singlefragment.html (accessed on 2 January 2022)). Using cDNA obtained by reverse transcription as a template, upstream and downstream primers were used to amplify the sequence of *PatNES*.

### 4.9. In Vitro Assays

For in vitro functional assays, the complete coding sequences of *PatNES* were cloned into the pET-28a vector and then digested with restriction enzymes *Bam*HI and *Xho*I. The products were transformed into *E. coli* based on the Trans5α Chemically Competent Cell protocol (www.transgen.com.cn (accessed on 29 January 2022)). The recombination plasmids were then transformed into Rosetta (DE3) competent cells following the protocol for Rosetta (DE3) Chemically Component Cells (WEIDI Bio, Qinghai, China). The positive colonies were cultured in LB medium with 25 μg/mL of chloramphenicol and kanamycin. Isopropyl β-D-thiogalactopyranoside (IPTG) was added to induce the expression of the protein (the final concentration of IPTG is 0.5 mM). Subsequently, the cell pellets were collected and resuspended in a balanced buffer (1 × PBS, 20 mM imidazole, pH 7.4) and sonicated on ice. The lysate from the samples was centrifuged at 7000 rpm and 4 °C for 30 min, and the clear lysate was then transferred to pre-washed Nickel NTA beads and incubated for 3 h at 4 °C. The Nickel NTA beads were then rinsed with 10 mL of a balanced buffer. The protein that was tagged was eluted by adding 3 mL of elution buffer (1 × PBS, 300 mM imidazole, pH 7.4). Finally, the proteins were desalted against a storage buffer (50 mM HEPES, pH 7.5) with PD-10 column. The purified proteins were identified using SDS-PAGE gel electrophoresis.

After purifying the protein, the reaction was conducted using the following system: purified protein 45 uL, FPP 5 uL, Activity Buffer 50 uL (50 mM HEPES, 5 mM MgCl_2_, 5 mM DTT, pH 7.5), and n-hexane 200 uL. The in vitro enzyme activation reaction was performed at 30 °C for 2 h. The products were detected with GC-MS (see details provided in the following section). The final compound was identified by searching the reference spectra in the database of Agilent Technologies and comparison of the retention time with the genuine standard (nerolidol).

### 4.10. Metabolite Analysis with GC-MS

The product of the in vitro enzyme activation reaction was detected using a Gas Chromatography-Mass Spectrometry spectrometer (Agilent Technologies, Santa Clara, CA, USA) on a column of Agilent 19091S-433UI (30.0 m × 250 μm × 0.25 μm) with an initial column temperature of 50 °C. The procedure lasts for 2 min, and then heated to 250 °C at 6 °C/min for 3 min. The scanning time started at 4.0 min and ended at 33.16665 min. The temperature of the vaporization chamber was 230 °C, and the injection volume was 1.0 μL. The carrier gas was pure helium with a flow rate of 1.0 mL/min and a pre-column pressure of 7.6522 psi. The mass spectrometry conditions were as follows: EI ion source, ion source temperature of 230 °C, the quadrupole temperature of 150 °C, the electron energy of 70 eV, an emission current of 34.6 μA, a multiplier voltage of 962 V, an interface temperature of 250 °C, and a mass range of 50–350 amu.

## 5. Conclusions

In this study, whole-genome resequencing was conducted on seven representative patchouli accessions, resulting in the detection of 402,650 high-quality SNPs and 153,233 high-quality INDELs. These variants provide valuable genetic resources for researchers and breeders of patchouli. Based on these genetic variants, the seven patchouli accessions have been classified into three groups: the Chinese group, the Southeast Asian group, and SP. Given the specificity of Shipai patchouli, 26,144 SNPs and 16,289 INDELs have been screened as its molecular markers, and 10 of them have been successfully verified. Furthermore, this study explores the genes that are affected by genetic variants in the sesquiterpene synthesis pathway. It has been demonstrated that the *PatNES* loses its nerolidol synthetase activity in patchouli accessions from Southeast Asia and SP. Our research has provided a glimpse into the diversity of patchouli germplasm resources and offers valuable information for functional genome research on patchouli.

## Figures and Tables

**Table 1:** Basic information for patchouli resequencing data.

ID	Raw Reads Number	Clean Reads Number	Duplication Rate (%)	Mean Depth (×)	Mean Coverage (%)	Mapped Ratio (%)	SNP Number	INDEL Number
YC	284,919,332	281,852,310	15.99	21.00	99.58	97.51	110,944	33,837
ZJ	286,562,660	284,505,424	14.94	21.80	99.55	99.87	95,858	29,126
HN	312,327,368	310,953,452	19.28	22.48	99.59	94.56	104,200	32,370
GY	275,765,722	273,674,814	16.21	20.67	99.53	98.41	103,142	30,697
PL	296,466,716	293,764,344	14.41	22.52	99.46	99.84	250,663	73,501
YN	272,246,368	270,794,548	16.51	19.69	99.54	95.05	248,053	66,545
SP	303,353,674	300,234,292	15.84	22.32	99.54	96.96	249,746	64,447

## Data Availability

The raw sequence data reported in this paper have been deposited in the Genome Sequence Archive [58] in National Genomics Data Center [59], China National Center for Bioinformation/Beijing Institute of Genomics, Chinese Academy of Sciences (GSA: CRA010718) that are publicly accessible at https://ngdc.cncb.ac.cn/gsa (accessed on 23 March 2023).

## Supplementary Materials

The following supporting information can be downloaded at: https://www.mdpi.com/article/10.3390/ijms241310970/s1.
