# Supplementary material for: Whole-Genome Resequencing Reveals the Diversity of Patchouli Germplasm
Source: Int J Mol Sci. 2023 Jun 30;24(13):10970. doi: 10.3390/ijms241310970 (PMC10341651; doi:10.3390/ijms241310970)
Supplement: Supplementary file 1 [file ijms-24-10970-s001.zip › supplementary informations.pdf]

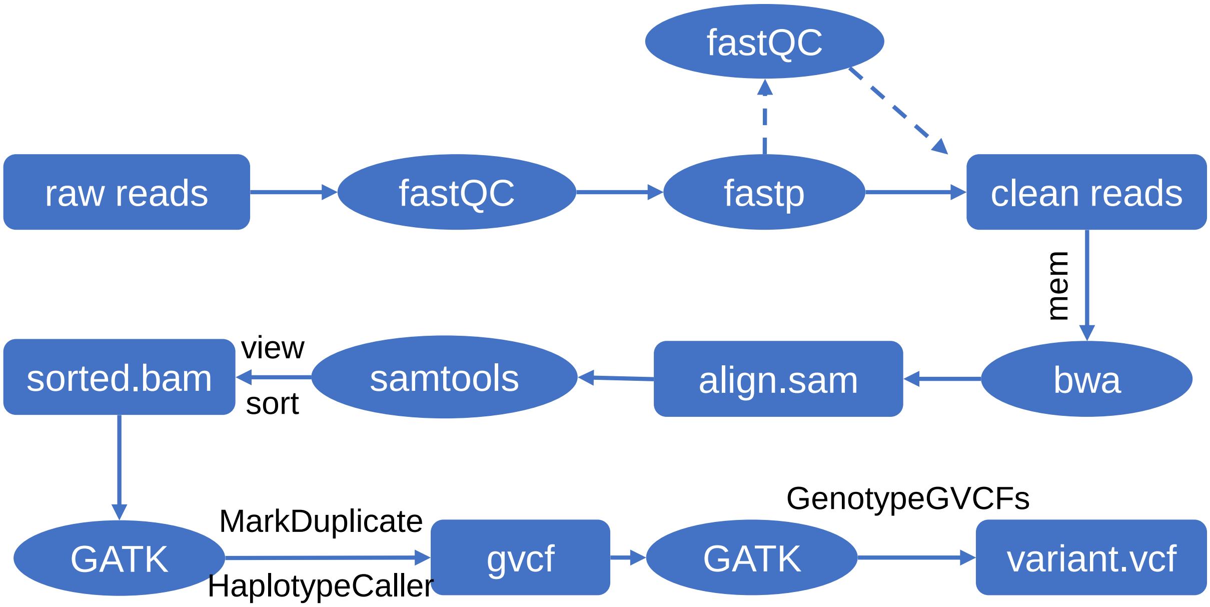

Supplementary Figure 1. Pipeline for genetic variation detection.

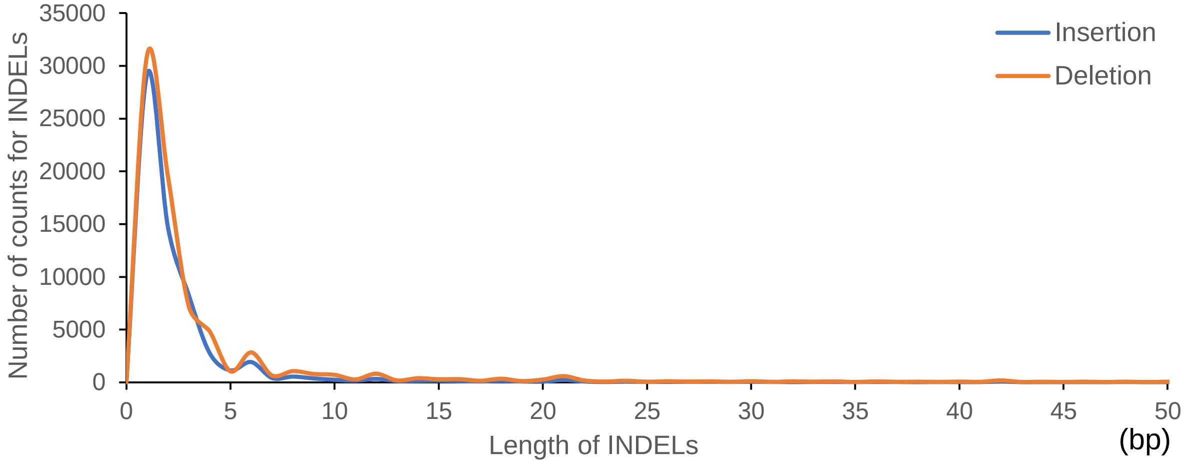

Supplementary Figure 2. Density curve of INDEL length

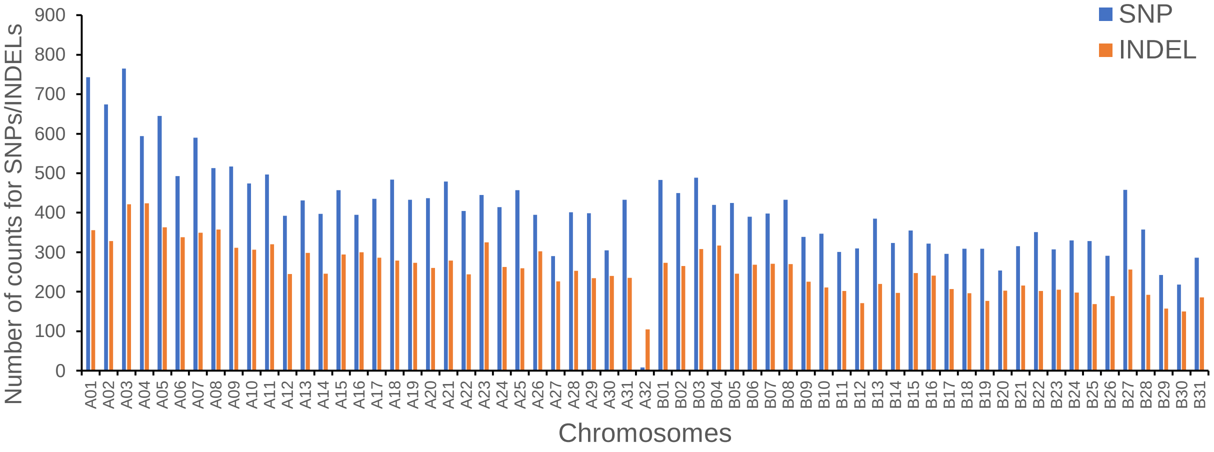

Supplementary Figure 3. Quantity distribution of SP specific SNPs and INDELs on chromosomes.

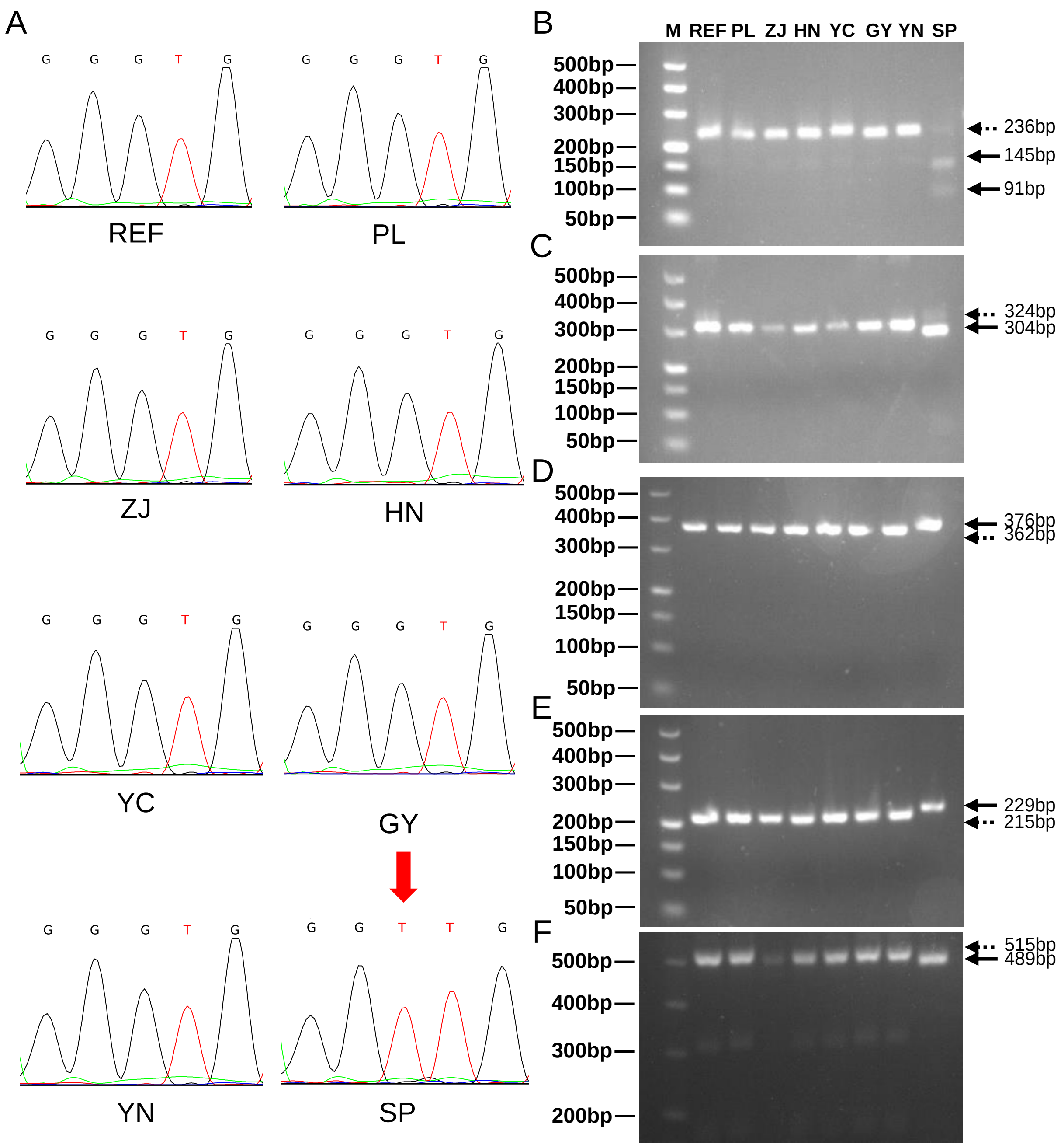

Supplementary Figure 4. Verification of molecular markers. (A) Sanger sequencing verification for SNP mutation of A20-14848597. (B) Enzymatic digestion verification for SNP of A02-38522213. (C-F) PCR directly amplifies the sequence for INDEL mutation of B04-23072200 (C), A15-14220318 (D), B09-16858306 (E) and B22-12443436 (F). REF represents the sequence of reference patchouli genome.

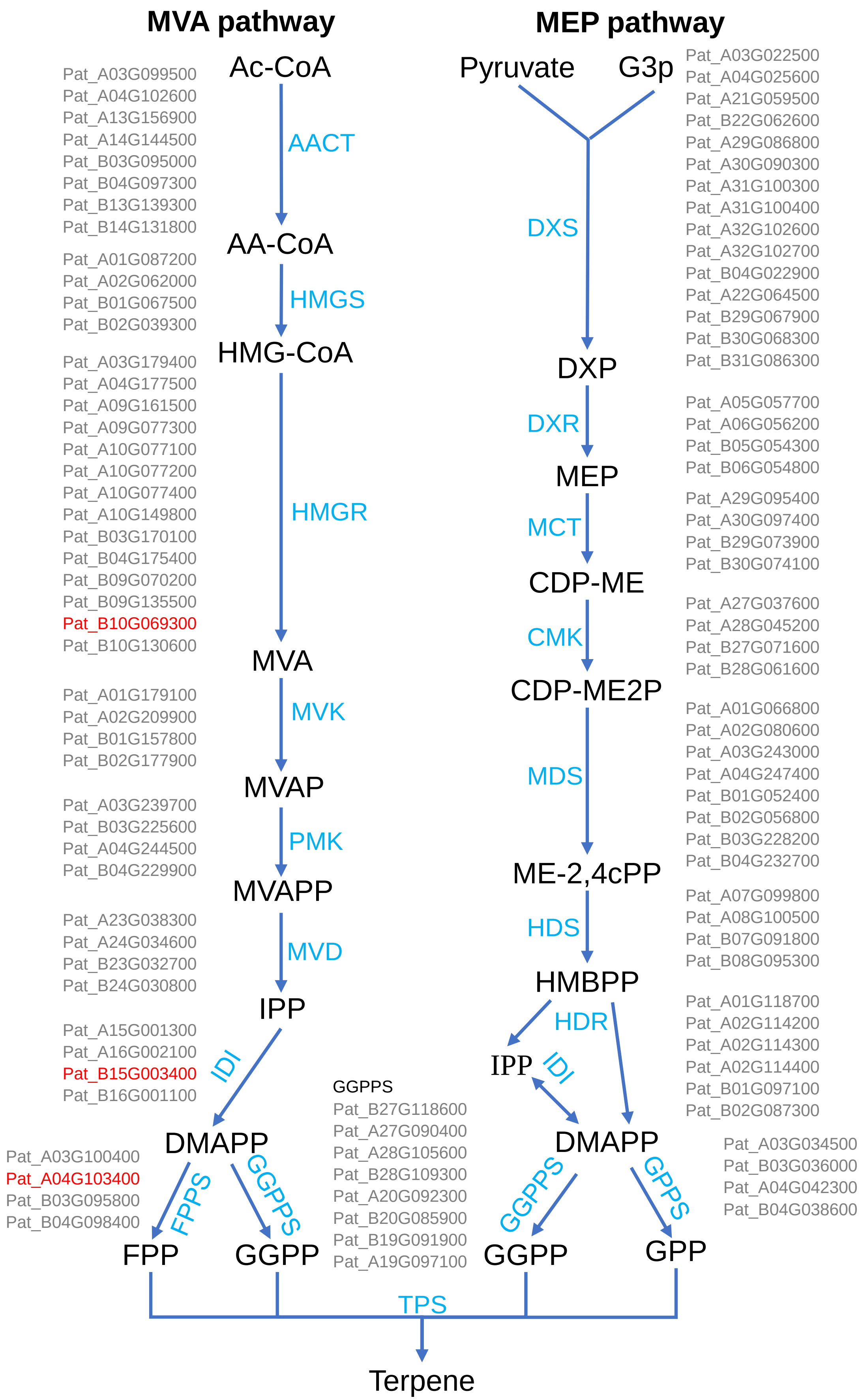

Supplementary Figure 5. Patchouli genes in the upstream of the sesquiterpene synthesis pathway. Red ID represents the gene affected by SNP/INDEL. Gray means the gene not affected.

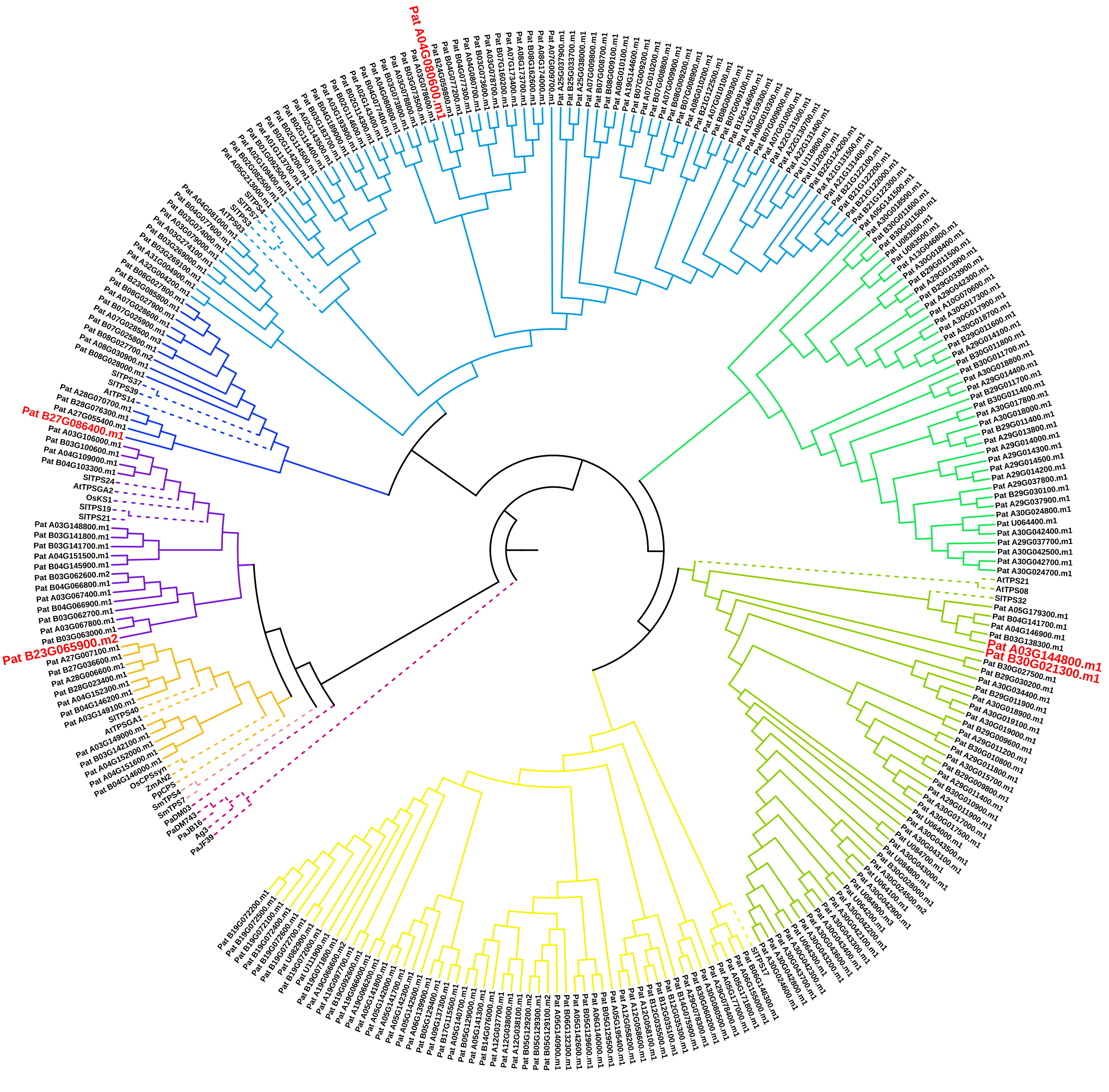

Supplementary Figure 6. TPS genes in patchouli genome. Red ID represents the gene affected by SNP/INDEL. Gray means the gene not affected.

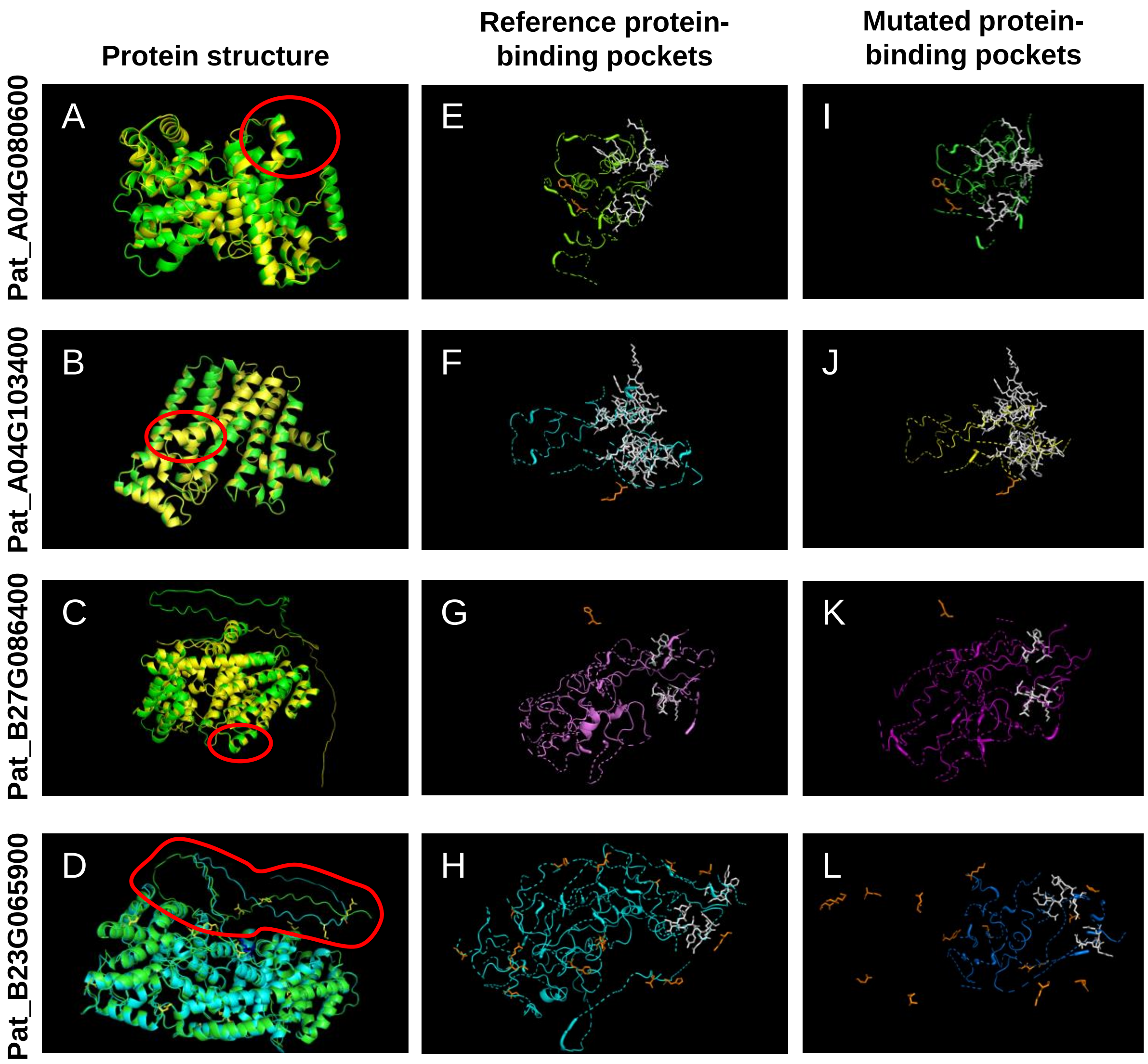

Supplementary Figure 7. The predicted three-dimensional structure and binding pockets of proteins of the 4 genes affected by genetic variants. In the protein structure (A-D), green represents unmutated protein structure while yellow or cyan represent mutated protein structure, the red circle is where the genetic variation mainly occurs. In the remainder figures (E-L), white amino acid residues are the conserve domain of proteins and orange amino acid residues are the mutation locus.

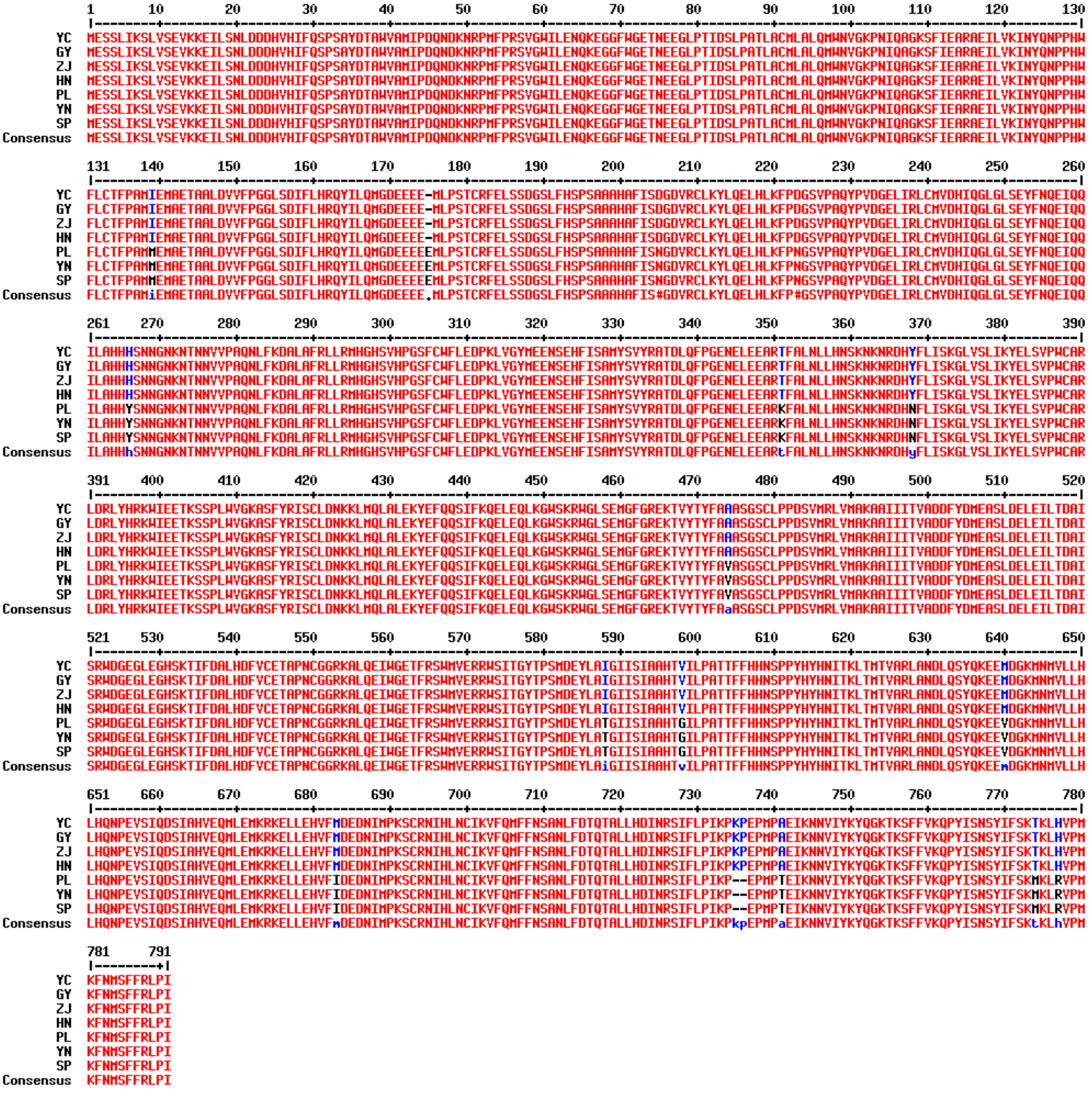

Supplementary Figure 8. The alignment of reference (REF) and mutated (ALT) *Pat_B23G065900* protein sequences.

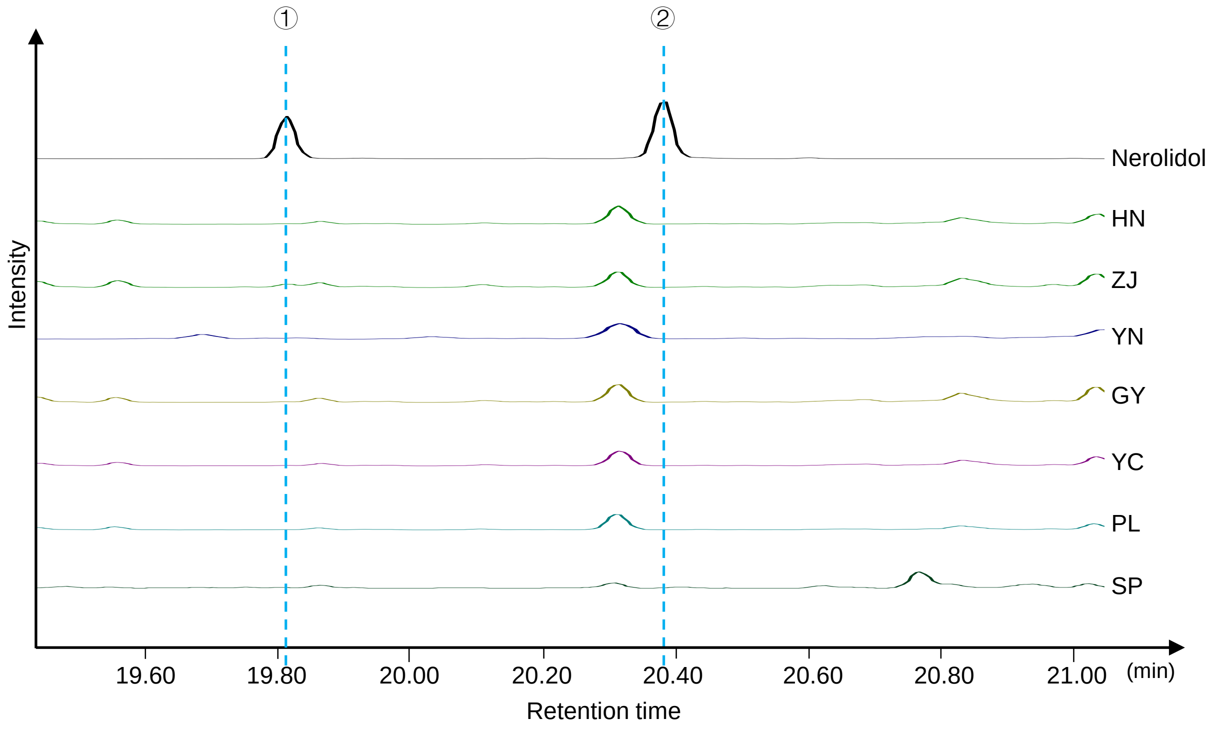

Supplementary Figure 9. GC-MS analysis of nerolidol on patchouli.

Both ① and ② are nerolidol (cis- and trans- isomers).
